# Systemic impact on secondary brain aggravation due to ischemia/reperfusion injury in post-cardiac arrest syndrome: a prospective observational study using high-mobility group box 1 protein

**DOI:** 10.1186/s13054-017-1828-5

**Published:** 2017-09-26

**Authors:** Atsunori Sugita, Kosaku Kinoshita, Atsushi Sakurai, Nobutaka Chiba, Junko Yamaguchi, Tsukasa Kuwana, Nami Sawada, Satoshi Hori

**Affiliations:** Division of Emergency and Critical Care Medicine, Department of Acute Medicine, Nihon University School of Medicine, 30-1 Oyaguchi Kamimachi, Itabashi-ku, Tokyo, 173-8610 Japan

**Keywords:** HMGB1, IL-6, NSE, SOFA score, Post-cardiac arrest syndrome, Systemic ischemia/reperfusion injury, Secondary brain injury

## Abstract

**Background:**

Ischemia/reperfusion injury (I/R) is an important pathophysiology of post-cardiac arrest syndrome (PCAS) against multiple organ dysfunction and mortality. The inflammatory response in PCAS causes systemic I/R. The purpose of this study was to demonstrate the pathophysiology of systemic I/R for secondary brain damage using the biomarkers high-mobility group box 1 (HMGB1), neuron-specific enolase (NSE), and interleukin-6 (IL-6).

**Methods:**

This study was designed as a single-institution prospective observational study. Subjects were observed for 90 days, and neurological outcome was classified according to the Glasgow-Pittsburgh Cerebral Performance Categories Scale (CPC). Serum HMGB1, NSE, and IL-6 were evaluated for variability, correlation with each biomarker, or the Sequential Organ Function Assessment (SOFA) score and CPC at return of spontaneous circulation at 0, 24, 48, and 168 h.

**Results:**

A total of 128 patients were enrolled in this study. Initial HMGB1 correlated with CPC (*ρ* = 0.27, *p* = 0.036) and SOFA score (*ρ* = 0.33, *p* < 0.001). The early phase of HMGB1 (0–24 h), all phases of IL-6, and the delayed phase of NSE (24–168 h) manifested poor neurological outcome. HMGB1 showed a significant correlation with NSE (*ρ* = 0.29, *p* = 0.002 at 0 h; *ρ* = 0.42, *p* < 0.001 at 24 h) and IL-6 (*ρ* = 0.36, *p* < 0.001 at 24 h).

**Conclusions:**

Serum HMGB1 for first 24 h after cardiac arrest was significantly correlated with SOFA score, NSE, and IL-6. This result suggests that systemic I/R may contribute to secondary brain aggravation. It is expected that research on HMGB1 focused on systemic I/R will help prevent aggravating neurological outcomes.

## Background

The inflammatory response in patients with post-cardiac arrest syndrome (PCAS) causes systemic ischemia/reperfusion injury (I/R), which may lead to multiple organ dysfunction and mortality, and is described as “sepsis-like syndrome” [1]. Inflammatory cytokines play an important role in systemic I/R and are promoted by high-mobility group box 1 protein (HMGB1) [2]. Previous pilot studies showed that elevated serum HMGB1 is related to poor neurological outcome in PCAS [3, 4]. However, this does not sufficiently explain how systemic I/R affects neurological outcome on the basis of the limited data available. HMGB1 protein is passively released by necrotic or damaged cells and actively secreted by innate immune cells. Extracellular HMGB1 promotes production of systemic inflammatory cells such as macrophages, monocytes, and dendritic cells [5]. HMGB1 is also regarded as a proinflammatory cytokine which act on systemic organ I/R [2]. I/R also causes oxidative stress on vital organs, such as the heart, liver, kidneys, and brain, accompanied by accumulation of HMGB1 [6]. Interleukin (IL)-6 is also activated by the inflammatory response to systemic I/R [7]. Several studies have shown that IL-6 is an inflammatory factor in PCAS [1, 8]. IL-6, which has an established measurement method, is known to be promoted by HMGB1. Consequently, IL-6 is considered to be useful in comparison with HMGB1. Early release of HMGB1 in brain tissue after brain ischemia has been reported [9] and is released into the extracellular space of brain tissue [10]. It is also related to increased permeability of the blood-brain barrier (BBB) [11]. In this context, serum concentration of brain proteins such as the neuron-derived enzyme neuron-specific enolase (NSE), which is released after stroke [12] and cardiac arrest [13], has been assessed as a method of predicting secondary brain injury. NSE is an established and well-known measurement method used to predict poor neurological outcome in PCAS. We speculate that these markers can identify possible cardiac arrest survivors and prognosis. We focused on systemic inflammation after cardiac arrest that induces whole-body ischemia, including the brain. The purpose of this study was to demonstrate the pathophysiology of systemic I/R due to secondary brain damage with analysis of the variability of three biomarkers (HMGB1, NSE, and IL-6) in an early phase of PCAS.

## Methods

### Study design

**Fig. 1:**
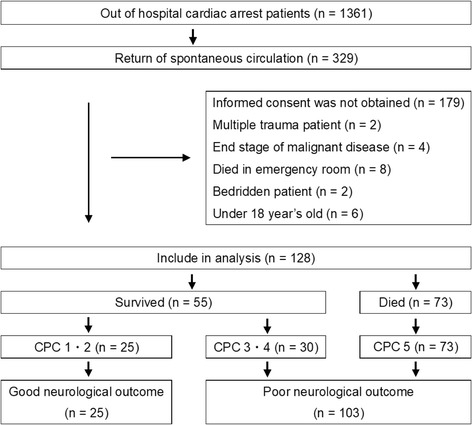
Flowchart of the study design and results of the number of patients. Outcome is shown at 90 days after return of spontaneous circulation. *CPC* Glasgow-Pittsburgh Cerebral Performance Categories Scale

This study was designed as a single-institution prospective observational study and was conducted from January 2011 to July 2013. Inclusion criteria were (1) patients who achieved return of spontaneous circulation (ROSC) from out-of-hospital cardiac arrest (OHCA) and who were brought to the emergency and critical care department, regardless of cardiac or noncardiac etiology; and (2) patients who did not meet the exclusion criteria. The exclusion criteria were (1) informed consent not obtained, (2) cases of multiple trauma, (3) end-stage malignancy, (4) death in the emergency room, (5) bedridden prior to hospitalization, and (6) less than 18 years old (Fig. 1).

Patients brought to the emergency and critical care department were registered and tabulated using the medical record and emergency medical service (EMS) report based on the Utstein style: (1) age, (2) sex, (3) witness present, (4) cardiopulmonary resuscitation (CPR) performed by a bystander, (5) shockable waveform observed on the electrocardiogram (ECG) at the time of EMS arrival, (6) time from receipt of the emergency call to ROSC, (7) adrenaline administration, and (8) cardiac etiology [14]. A shockable waveform is a wave pattern on the ECG that indicates arrhythmia necessitating defibrillation, such as ventricular fibrillation or pulseless ventricular tachycardia.

The following factors related to treatment and evaluation after hospitalization were also considered: (1) whether coronary angiography and percutaneous coronary intervention (PCI) were performed and (2) whether therapeutic hypothermia (TH) was performed. Three biomarkers (HMGB1, IL-6, and NSE) were compared. The primary endpoint was set as neurological outcome at 90 days after ROSC.

### Clinical protocol

Basic life support was performed by public and co-medics, including use of an automated external defibrillator. Defibrillation, airway protection including intubation, and adrenaline infusion were performed by EMS personnel [15].

Advanced cardiovascular life support [16, 17] and emergency cardiovascular care [18, 19] were performed prior to admission to the intensive care unit (ICU). TH was performed according to the following exclusion criteria: (1) unstable hemodynamics even with the use of vasopressors (mean blood pressure < 60 mm Hg or systolic blood pressure < 90 mm Hg), (2) inadequate oxygenation (ratio of partial pressure of arterial oxygen to fraction of inspired oxygen < 200), (3) end stage of a chronic disease, and (4) informed consent not obtained from the patient’s family [20]. Patients were subjected to a modulated temperature of 34 °C for 24 h during TH and gradually rewarmed from 34 °C to 36 °C for 24 h using an external cooling device. During TH, patients were placed under anesthesia using midazolam hydrochloride as sedation, fentanyl citrate as analgesia, and rocuronium bromide as a muscle relaxant.

### Sample collection

Physical findings and clinical measurements were recorded in the medical chart. Blood samples collected from the peripheral artery were evaluated using clinical variables within 6 h of ROSC and 24 h, 48 h, and 7 days after ROSC (abbreviated as 0 h, 24 h, 48 h, and 168 h, respectively). Serum samples for biomarkers (HMGB1, IL-6, and NSE) were centrifuged at 3000 cycles for 15 minutes at 4 °C. Separated samples were preserved frozen in a −80 °C refrigerator until analysis. Blood parameters were measured in the clinical laboratory department by coagulation test, chemical examination, and complete cell count.

### Measurements

Serum concentration of HMGB1 was measured using an enzyme-linked immunosorbent assay (ELISA; Shino-Test Corporation, Kanagawa, Japan). The lower sensitivity of HMGB1 was 0.2 ng/ml, and the cross-reactivity of HMGB2 was < 2%. IL-6 and NSE were measured using the Quantikine® ELISA kit (R&D Systems, Minneapolis, MN, USA). The minimum detectable doses of IL-6 and NSE were typically < 0.70 pg/ml and < 0.038 ng/ml, respectively.

### Mortality, multiple organ dysfunction, and outcome

Survival time and mortality in the early phase of PCAS was evaluated. Multiple organ dysfunction was evaluated according to individual organ dysfunction subscales of the Sequential Organ Failure Assessment (SOFA) [21]. SOFA scores were measured at ROSC in the early phase of PCAS according to (1) Glasgow Coma Scale (GCS) score, (2) blood pressure with vasopressor, (3) platelet count, (4) total bilirubin, and (5) creatinine. To rule out the influence of first brain insult by cardiac arrest, SOFA scores excluding GCS scores between two neurological outcome groups were compared.

Neurological outcome was evaluated according to the Glasgow-Pittsburgh Cerebral Performance Categories Scale (CPC) as follows: CPC 1 (good recovery), CPC 2 (moderate disability), CPC 3 (severe disability), CPC 4 (vegetative state), and CPC 5 (death). Subjects were divided into two groups by CPC category: a good neurological outcome group (CPC 1 or 2) and a poor neurological outcome group (CPC 3–5).

### Biomarkers, multiple organ dysfunction, and neurological outcome

SOFA score was evaluated according to CPC and HMGB1 at 0 h. The variability of three biomarkers (HMGB1, IL-6, and NSE) was assessed, and each peak level was compared. Serum levels of the three biomarkers were compared between the two neurological outcome groups, and correlations were evaluated.

### Subanalysis

In this study, patients were divided into groups for subgroup analysis, namely good or poor neurological outcome, cardiac etiology or noncardiac etiology, and with PCI or without PCI, in order to assess biomarker variability at various points in time.

### Statistical analysis

Statistical analysis was performed using the IBM SPSS Statistics version 22 statistical software program (IBM, Armonk, NY, USA). The Kaplan-Meier method was performed to evaluate mortality. Collected measurements were analyzed for normal distribution using the Shapiro-Wilk test. Median and IQR (first quartile to third quartile) statistics were used for nonparametric measurements, and average and SD were used for parametric measurements.

The Mann-Whitney *U* test was performed for nonparametric data, and Student’s *t* test was performed for parametric data. The chi-square test and Fisher’s exact test were performed for categorical data. The Kruskal-Wallis test was performed for multiple data comparisons. Spearman’s rank correlation test was performed to evaluate correlations. Simple logistic regression analysis was carried out. An explanatory variable was set as HMGB1 at 0 h, and response variables included two neurological outcome groups.

## Results

### Patient characteristics

A total of 128 patients were enrolled in this study after the exclusion criteria were applied. Of 1361 patients with OHCA who were brought to the emergency room, 329 patients achieved ROSC. Among these of 329 patients, 201 satisfied the exclusion criteria, including informed consent not being obtained (*n* = 179), cases of multiple trauma (*n* = 2), end-stage malignancy (*n* = 4), death in the emergency room (*n* = 8), bedridden prior to hospitalization (*n* = 2), and being less than 18 years old (*n* = 6).

Median survival time was 25 days, and the worst mortality was 11% within the first 24 h. Survival numbers and times are as follows: 114 after 24 h, 101 after 48 h, 82 after 7 days, and 55 after 90 days. In total, 73 patients died within the follow-up interval of 90 days. Twenty-five patients had a good neurological outcome (CPC 1, *n* = 24; CPC 2, *n* = 1), and 103 patients had a poor neurological outcome (CPC 3, *n* = 13; CPC 4, *n* = 17; CPC 5, *n* = 73). Seventy-two patients classified as CPC 5 (*n* = 73) died without recovery from the comatose state even once within the follow-up period. One patient classified as CPC 5 recovered from the comatose state but died within 90 days as a result of cardiac complications.

Characteristics of all patients are shown in Table 1. Seventy-three (57%) patients had a cardiac etiology, and 55 (43%) had a noncardiac etiology. The ratio of patients with a cardiac etiology was higher in the good neurological outcome group. Noncardiac etiology included airway obstruction due to a foreign body (*n* = 19), acute stroke (*n* = 7), pneumonia (*n* = 7), chronic obstructive pulmonary disease (*n* = 4), hyperkalemia (*n* = 4), gastrointestinal hemorrhage (*n* = 3), neck hanging (*n* = 3), sepsis (*n* = 3), drug intoxication (*n* = 1), diabetic ketoacidosis (*n* = 1), heatstroke (*n* = 1), and pulmonary embolus (*n* = 1).

**Table 1:** Characteristics of all patients according to neurological outcome 90 days after return of spontaneous circulation

	CPC 1 and 2 (*n* = 25)	CPC 3–5 (*n* = 103)	*p* Value
CPC 90 days after ROSC, *n* (%)			
1	24 (96)	0 (0)	
2	1 (4)	0 (0)	
3	0 (0)	13 (12.6)	
4	0 (0)	17 (16.5)	
5	0 (0)	73 (70.9)	
Background			
Cardiac etiology, *n* (%)	23 (92)	50 (49)	0.001
Age, median (IQR)	60 (48–71)	72 (63–83)	0.001
Male sex, *n* (%)	24 (96)	64 (62)	0.001
Witness, *n* (%)	18 (72)	72 (70)	1.00
Bystander CPR, *n* (%)	14 (56)	37 (36)	0.073
Initial shockable wave, *n* (%)	17 (68)	30 (29)	0.001
Time interval from emergency call to ROSC, minutes, mean ± SD	28.4 ± 22.3	46.7 ± 25.0	<0.001
Adrenaline administration, mg, median (IQR)	0 (0–1)	2 (1–3)	0.001
PCI performed, *n* (%)	13 (52)	21 (20)	0.002
Therapeutic hypothermia performed, *n* (%)	17 (68)	53 (51)	0.18
Required vasopressor, yes, *n* (%)	7 (28)	77 (75)	<0.001
SOFA score			
SOFA on admission, mean ± SD	8.1 ± 2.6	10.1 ± 2.6	0.001
SOFA excluding GCS, mean ± SD	4.2 ± 2.7	6.1 ± 2.5	0.001
GCS	3.9 ± 0.3	4.0 ± 0.1	0.223
PaO_2_/FiO_2_ ratio	2.4 ± 1.6	2.7 ± 1.4	0.448
Circulation	1.0 ± 1.4	2.2 ± 1.4	<0.001
Total bilirubin	0 ± 0.0	0.1 ± 0.3	0.011
Creatinine	0.7 ± 1.1	0.7 ± 1.1	0.94
Platelet	0.7 ± 0.3	0.5 ± 0.7	0.001

*Abbreviations: ROSC* Return of spontaneous circulation, *CPC* Glasgow-Pittsburgh Cerebral Performance Categories Scale, *CPR* Cardiopulmonary resuscitation, *PCI* Percutaneous coronary Intervention, *TH* Therapeutic hypothermia, SOFA Sequential Organ Failure Assessment, *PaO*_*2*_ */FiO*_*2*_ Ratio of partial pressure of arterial oxygen to fraction of inspired oxygen, *GCS* Glasgow Coma Scale

*Required vasopressor* refers to patients who needed an intravenous vasopressor after initial resuscitation on the first hospital day. Parametric data are presented as mean ± SD. Nonparametric data are presented as median with IQR. Categorical data are presented as *n* (%). The percentages in parentheses are the ratios for poor outcome or good outcome. Statistical significance was set at *p* < 0.05

A significant difference in sex and age was observed, but witness and bystander CPR were similar in the two groups. Patients in the poor neurological outcome group had a significantly longer interval from receipt of the emergency call to ROSC, as well as to nonshockable wave, and a higher dose of required adrenaline administration than patients in the good neurological outcome group.

Emergency cardiac angiography was performed on 63 patients. Patients in the good neurological outcome group achieved a higher PCI than those in the poor neurological outcome group. TH was performed on 70 (55%) patients and was similar between the two groups. Correlation with SOFA score was significant in the poor neurological outcome group, especially for circulation instability, liver dysfunction, and coagulation dysfunction (Table 1).

### Biomarker variability

**Fig. 2:**
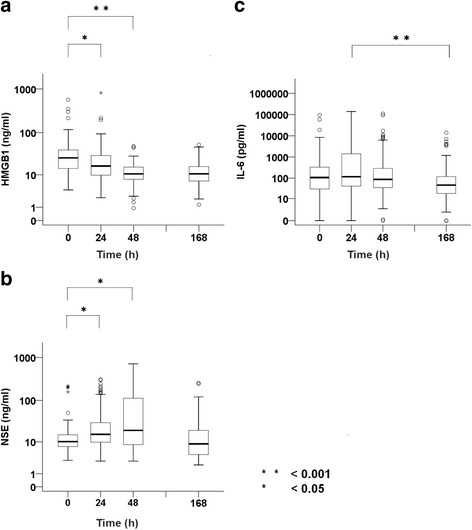
Variability of biomarkers (high-mobility group box 1 protein [HMGB1], neuron-specific enolase [NSE], and interleukin [IL]-6) from return of spontaneous circulation (ROSC). Biomarker variability was analyzed from ROSC to 7 days using box plots for HMGB1 (**a**), NSE (**b**), and IL-6 (**c**). Measurement points were at 0, 24, 48, and 168 h (7 days) after ROSC. This plot is presented on a logarithmic scale. Statistical significance was set at *p* < 0.05 (*) and *p* < 0.001 (**) in the box plot. This box plot consists of *boxes*, *whiskers*, *open circles*, and *asterisks* using a logarithmic scale. The *horizontal bold line* in the middle of the box is the median value. The *box* is the IQR from the first quartile to the third quartile. *Whiskers* are the range of maximum and minimum values between 1.5 times IQR above the third quartile and 1.5 times IQR below the first quartile. *Open circles* are the outliers between 1.5 and 3 times IQR either above the third quartile or below the first quartile. *Asterisks* are the outliers three times the IQR either above the third quartile or below the first quartile. Number of patients in the graphs are 0 h (*n* = 128), 24 h (*n* = 114), 48 h (*n* = 101), and 168 h (*n* = 82)

A significant sequential difference in the levels of initial serum HMGB1 or NSE after ROSC was observed. When comparing values at 0 h with those at 24 or 48 h, we observed a gradual decrease (HMGB1; Fig. 2a) or increase (NSE; Fig. 2b). However, there was no significant change in serum HMGB1 or NSE levels between 48 and 168 h (Fig. 2a, b). Regarding IL-6 levels, no significant difference was observed for ROSC at 24, 48, or 168 h (Fig. 2c).

SOFA score was positively and significantly correlated with CPC (*ρ* = 0.44, *p* < 0.001) and HMGB1 (*ρ* = 0.33, *p* < 0.001) at 0 h. A significant positive correlation between HMGB1 and neurological outcomes (CPC 1, 2, 3, 4, or 5) was observed, and a high HMGB1 indicated a weak but significant correlation with a poor neurological outcome (ρ = 0.27, *p* = 0.036). The relationships between HMBG1 and IL-6 or NSE at the same point in time indicated that HMGB1 had a partial but significant positive correlation with NSE (0 h, *ρ* = 0.29, *p* = 0.002; 24 h, *ρ* = 0.42, *p* < 0.001; 48 h, *ρ* = 0.17, *p* = 0.135) and IL-6 (0 h, *ρ* = 0.14, *p* = 0.126; 24 h, *ρ* = 0.36, *p* < 0.001; 48 h, *ρ* = 0.11, *p* = 0.337).

### Biomarkers and outcome

**Fig. 3:**
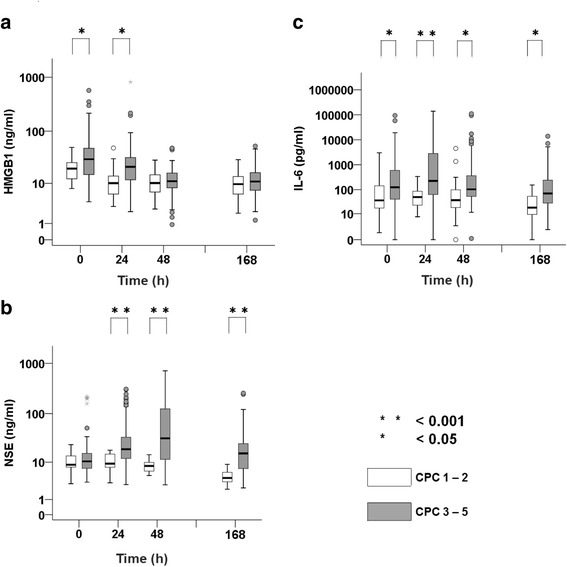
Box plot comparing biomarkers by outcome. Mann-Whitney *U* test was performed. Analysis results are shown by a box plot in a logarithm scale. Serum level of high-mobility group box 1 protein (HMGB1) (**a**), neuron-specific enolase (NSE) (**b**), and interleukin (IL)-6 (**c**) were compared with group Glasgow-Pittsburgh Cerebral Performance Categories Scale (CPC) 3–5 and CPC 1 or 2 at 0, 24, 48, and 168 h (7 days). Statistical significance was set at *p* < 0.05 (*) and *p* < 0.001 (**) above the box plot. The *horizontal bold line* in the middle of the box is the median value. The *box* is the IQR from the first quartile to the third quartile. *Whiskers* are the range of maximum and minimum values between 1.5 times IQR above the third quartile and 1.5 times IQR below the first quartile. *Open circles* are the outliers between 1.5 and 3 times IQR either above the third quartile or below the first quartile. *Asterisks* are the outliers three times IQR either above the third quartile or below the first quartile. Number of patients in the graphs (**a**–**c**) are 0 h (*n* = 25), 24 h (*n* = 25), 48 (*n* = 25), and 168 (*n* = 25) for CPC 1 or 2; and 0 h (*n* = 103), 24 h (*n* = 89), 48 h (*n* = 76), and 168 h (*n* = 57) for CPC 3 or 4

The correlation between biomarkers and patient outcome is shown in Fig. 3. Serum HMGB1 (Fig. 3a) was significantly higher in the poor neurological outcome group than in the good neurological outcome group at 0 and 24 h. In the poor neurological outcome group, serum NSE (Fig. 3b) and IL-6 (Fig. 3c) values were significantly higher, with a wide dispersion, than those in the good neurological outcome group from 0 to 168 h, but there was no difference between these outcome groups for NSE at 0 h. Serum NSE was similar in the two groups at 0 h and began to increase after 24 h. In the poor neurological outcome group, NSE peaked with a median value at 48 h, later than HMGB1 and IL-6 peaked.

**Fig. 4:**
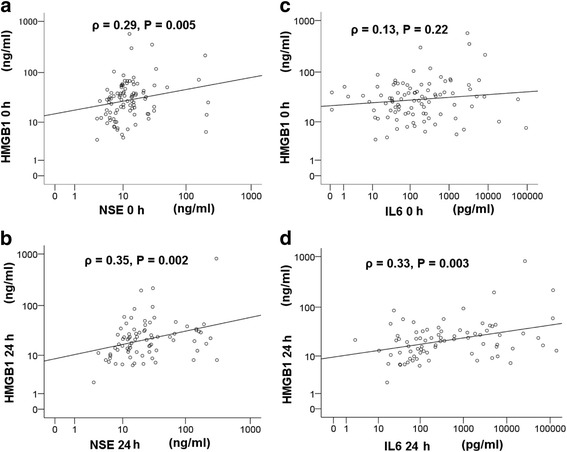
Scatterplot showing the correlation between high-mobility group box 1 protein (HMGB1) and neuron-specific enolase (NSE) or interleukin (IL)-6 in the poor outcome group. Spearman’s rank correlation test was performed to analyze the correlation between HMGB1 and NSE or IL-6 in the poor outcome group. Correlation between HMGB1 and NSE at return of spontaneous circulation (ROSC) (**a**) and 24 h after ROSC (**b**) is represented on a logarithmic scale. Correlation between HMGB1 and IL-6 at ROSC (**c**) and 24 h after ROSC (**d**) is represented on a logarithmic scale. The measurement points at ROSC and 24 h are represented as 0 h and 24 h, respectively, in this graph. The coefficient of correlation is shown by ρ, and *p* < 0.05 is defined as statistically significant above the scatterplot. The reference line indicates positive correlation. The numbers of patients represented in the graphs are *n* = 103 (**a**), *n* = 89 (**b**), *n* = 103 (**c**), and *n* = 89 (**d**)

On the basis of single-variable logistic regression analysis, a significant poor outcome for PCAS was identified by observing good neurological outcomes in patients with low HMGB1 (adjusted OR 0.963, 95% CI 0.933–0.994, *p* = 0.021) according to changes in each single value. Of these patients, a significant correlation was observed between HMGB1 and NSE or IL-6 at 0 h (NSE, *ρ* = 0.29, *p* = 0.002; IL-6, *ρ* = 0.14, *p* = 0.126) or 24 h (NSE, *ρ* = 0.42, *p* < 0.001; IL-6, *ρ* = 0.36, *p* < 0.001). In the subanalysis of this study, no significant correlation between HMGB1 and NSE at 0 h (*ρ* = 0.089, *p* = 0.680), 24 h (*ρ* = −0.13, *p* = 0.586), or 48 h (*ρ* = 0.30, *p* = 0.195) in the good neurological outcome group was observed. However, a significant positive correlation between HMGB1 and NSE at 0 h (*ρ* = 0.29, *p* = 0.005) and 24 h (*ρ* = 0.35, *p* = 0.002) was observed, but not at 48 h, in the poor neurological outcome group (Fig. 4).

**Fig. 5:**
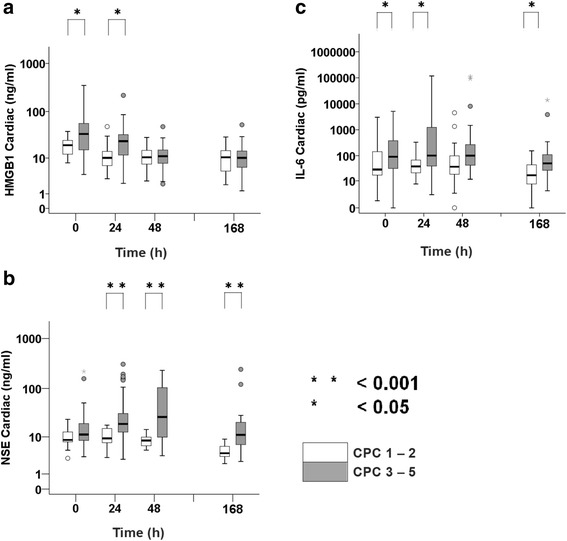
Box plot comparing biomarkers by outcome in cardiac etiology. The Mann-Whitney *U* test was performed. Analysis results are shown by box plot on a logarithmic scale. Serum level of high-mobility group box 1 protein (HMGB1) (**a**), neuron-specific enolase (NSE) (**b**) and interleukin (IL)-6 (**c**) were compared with group Glasgow-Pittsburgh Cerebral Performance Categories Scale (CPC) 3–5 and CPC 1 or 2 at 0, 24, 48, and 168 h (7 days) in cardiac etiology for subanalysis. Statistical significance was set at *p* < 0.05 (*) and *p* < 0.001 (**) above the box plot. The *horizontal bold line* in the middle of the box is the median value. The *box* is the IQR from the first quartile to the third quartile. *Whiskers* are the range of maximum and minimum values between 1.5 times IQR above the third quartile and 1.5 times IQR below the first quartile. *Open circles* are the outliers between 1.5 and 3 times IQR either above the third quartile or below the first quartile. *Asterisks* are the outliers three times the IQR either above the third quartile or below the first quartile. Numbers of patients in the graphs are 0 h (*n* = 23), 24 h (*n* = 23), 48 h (*n* = 23), 168 h (*n* = 23) for CPC 1 or 2; and 0 h (*n* = 50), 24 h (*n* = 45), 48 h (*n* = 39), 168 h (*n* = 27) for CPC 3 or 4

However, the correlation between neurological outcome and biomarkers (HMGB1, NSE, and IL-6) according to cardiac etiology (Fig. 5) was similar to that of cases that included cardiac and noncardiac etiology.

**Fig. 6:**
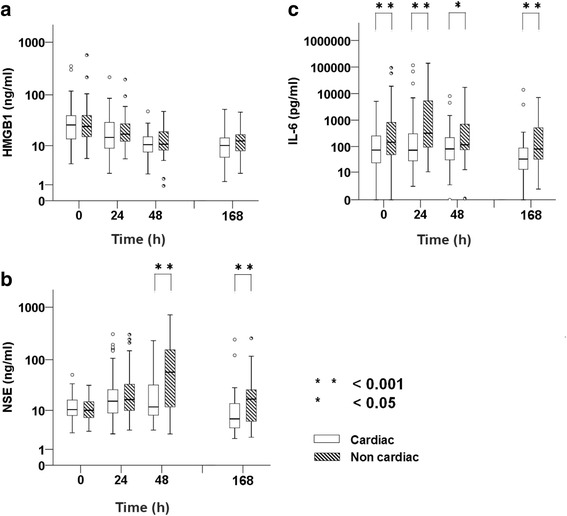
Box plot comparing biomarkers by cardiac etiology or noncardiac etiology. The Mann-Whitney *U* test was performed. Analysis results are shown by a box plot on a logarithmic scale. Serum levels of high-mobility group box 1 protein (HMGB1) (**a**), neuron-specific enolase (NSE) (**b**), and interleukin (IL)-6 (**c**) were compared with cardiac etiology and noncardiac etiology at 0, 24, 48, and 168 h (7 days). Statistical significance was set at *p* < 0.05 (*) and *p* < 0.001 (**) above the box plot. The *horizontal bold line* in the middle of the box is the median value. The *box* is the IQR from the first quartile to the third quartile. *Whiskers* are the range of maximum and minimum values between 1.5 times IQR above the third quartile and 1.5 times the IQR below the first quartile. *Open circles* are the outliers between 1.5 and 3 times the IQR either above the third quartile or below the first quartile. *Asterisks* are the outliers three times the IQR either above the third quartile or below the first quartile. Numbers of patients in the graphs are 0 h (*n* = 73), 24 h (*n* = 68), 48 h (*n* = 62), 168 h, (*n* = 50) in the cardiac group; and 0 h (*n* = 55), 24 h (*n* =46), 48 h (*n* = 39), 168 h (*n* = 32) in the noncardiac group

Serum biomarkers according to cardiac or noncardiac etiology demonstrated that serum HMGB1 was similar between the two patterns of etiology, but NSE and IL-6 increases were higher in noncardiac etiology cases than in cardiac etiology cases (Fig. 6).

With PCI or without PCI, serum HMGB1 levels (median [IQR]) did not show a significant difference for with PCI vs without PCI (0 h, 24.0 [12.5 – 34.7] vs 28.9 [12.6 – 54.6], *p* = 0.56; 24 h, 16.1 [9.86 – 32.2] vs 12.9 [8.5 – 27.7], *p* = 0.75).

## Discussion

Some pilot studies focused on HMGB1 after cardiac arrest have been done regarding the influence on neurological outcome [3, 4]. However, the pathophysiology of elevated serum HMGB1 affecting poor neurological outcome is not sufficiently clear. The correlation between systemic I/R and secondary brain aggravation has not been discussed. In the present study, serum HMGB1 correlated with serum NSE in patients with PCAS. This phenomenon may indicate a possible mechanism of a secondary brain injury process with systemic I/R after cardiac arrest.

Increasing serum HMGB1 at ROSC means that initial HMGB1 was passively released by necrotic or damaged cells due to cardiac arrest. The increase of HMGB1 affecting the neurological outcome lasts 24 h and shows a correlation with IL-6, indicating an inflammatory response. Serum elevation of HMGB1 for the first 24 h may include active excretion by inflammatory cells. These two patterns of pathways to release of HMGB1 may promote exacerbation to inflammation as a systemic I/R in PCAS. The correlation with HMGB1 and SOFA score indicates that excessive inflammation in the early phase of PCAS contributes to the organ damage.

Interestingly, our study indicates a positive correlation between serum HMGB1 and NSE in the early phase, which is correlated with neurological outcome. This phenomenon was significantly observed in patients with a poor neurological outcome, but not in patients with a good neurological outcome.

The molecular weights of NSE and HMGB1 are approximately 80,000 Daltons [22] and 30,000 Daltons [3], respectively. Systemic I/R, including in the brain, may lead to NSE or HMGB1 leaking into the cerebrospinal fluid and systemic blood [11], increasing the permeability of the BBB [23]. Recent studies have shown that HMGB1, not only in cerebrospinal fluid but also in blood, can induce a brain inflammatory response and contribute to brain injury [24, 25]. Although these biomarkers have limited ability to cross the BBB, the inflammatory response in the brain is thought to relate to neurological outcome after post-cardiac arrest hypoxia, and brain inflammation as a secondary aggravation process may result from systemic I/R due to the change in the ability to cross the BBB after cerebral ischemia [26, 27]. IL-6 may also affect infiltration of inflammatory cells and induce organ damage. These systemic inflammations may play an important role in the postinflammatory effect on systemic organ damage, including brain tissue, as estimated by SOFA score [28]. These conditions might also be related to serum HMGB1 elevation and neurological outcome in PCAS.

It is undeniable that an inflammatory response after resuscitation comes from an etiology before cardiac arrest. In subanalysis, serum elevation of HMGB1, IL-6, and NSE contributed to neurological outcome in the case of cardiac etiology, which has less influence on inflammation and brain injury than a noncardiac etiology. A sequential response of post-cardiac arrest including HMGB1 can be observed in common, regardless of cardiac or noncardiac etiology.

Serum HMGB1 has been reported to be independently associated with increased mortality in patients with ST elevation myocardial infarction treated with PCI [29]. Regarding serum level of HMGB1, a significant difference between patients receiving PCI and patients not receiving PCI was not observed in our study. This might indicate that the cause of increased HMGB1 includes other factors (whole-body ischemia including the brain) in PCAS.

Most physicians consider whole brain anoxia/hypoxia as a major pathogenesis of poor neurological outcome in PCAS. Although the main cause of poor neurological outcome is the primary anoxic brain injury, the length of time until ROSC after cardiac arrest may be the most important factor for prediction of final outcome [30, 31]. However, the question remains whether the secondary brain injury process after ROSC influences neurological outcome in patients with PCAS. Systemic I/R, cardiac dysfunction, and persistent pathophysiology, in addition to primary anoxia/hypoxia brain injury after cardiac arrest, should be considered in the pathogenesis of PCAS to poor neurological outcome [32]. Treatment focusing on I/R after cardiac arrest is not considered, although target temperature management [33], including with brain hypothermia, may be effective for systemic inflammation after cardiac arrest [34]. Because the main treatment goal for patients with PCAS is focused on a secondary brain injury process, our results suggest that the next step in treatment strategy should be consideration of systemic I/R. On the basis of these results, a correlation between neurological outcome of PCAS and early systemic inflammatory response leading to exacerbation of inflammatory balance [35] is suggested and may possibly be associated with secondary brain injury processes after systemic I/R.

Although it is unclear whether the origin of serum HMGB1 is from a peripheral organ or brain tissue, NSE is mainly considered to be of brain origin [36]. If HMGB1 increases in the brain extracellular space after ROSC with an injured and disturbed BBB and brain autoregulation caused by a more severe I/R [37], brain damage can be further aggravated during the injury processes. Taking this information together, we speculate that an aggravation of brain injury processes in patients with PCAS could be estimated by measuring serum HMGB1 in the early phase of PCAS, preceding the upregulation of proinflammatory cytokines.

This study has some limitations. Healthy volunteers did not participate in this study. Normal levels of the biomarkers were not measured, and the contribution of primary brain hypoxia on neurological outcome was not evaluated in the small number of patients. The origin of HMGB1 remains unclear because the mediators could have passed through the BBB after brain vascular permeability was altered.

In addition to NSE, S100β and glial fibrillary acidic protein (GFAP) are known as biomarkers reflecting brain damage in PCAS. S100β predicts poor neurological outcome with NSE. S100β has a short half-life of about 30 minutes: The serum level rapidly decreases within 1 h [38], and thus it was not suitable for long-term measurement in our study (this study followed 7 days). However, the predictive outcome value of GFAP is not established [39, 40]. Because S100β and GFAP were not selected for analysis, glial disintegration by cardiac arrest was not assessed in this study.

Increased serum HMGB1 does not show high specificity in PCAS. The influence of serum HMGB1 before and after cardiac arrest could not be completely excluded. However, a significant difference between cardiac arrest etiologies (cardiac or noncardiac) was not observed.

In this study, increasing HMGB1 correlated with SOFA score and poor neurological outcome in the early phase. HMGB1 is, however, reported to play a role in repairing damaged tissue as well as promoting inflammation [41]. Because the beneficial aspect of regeneration by HMGB1 was not analyzed in this study, whether inhibiting HMGB1 would mitigate tissue damage remains unknown.

## Conclusions

Our study indicates that serum HMGB1 for first 24 h after cardiac arrest significantly correlates with SOFA score, NSE, and IL-6. This result was observed in the poor neurological outcome group, which shows that systemic I/R after cardiac arrest may contribute to secondary brain aggravation, depending on the severity with increasing BBB permeability. It is expected that research on HMGB1 focusing on systemic I/R will help to prevent aggravating neurological outcome.

## Abbreviations

BBB: Blood-brain barrier
CPC: Glasgow-Pittsburgh Cerebral Performance Categories Scale
CPR: Cardiopulmonary resuscitation
ECG: Electrocardiogram
ELISA: Enzyme-linked immunosorbent assay
EMS: Emergency medical service
GCS: Glasgow Coma Scale
GFAP: Glial fibrillary acidic protein
HMGB1: High-mobility group box 1 protein
I/R: Ischemia/reperfusion injury
ICU: Intensive care unit
IL-6: Interleukin-6
IQR: Interquartile range
NSE: Neuron-specific enolase
OHCA: Out-of-hospital cardiac arrest
PaO_2_/FiO_2_: Ratio of partial pressure of arterial oxygen to fraction of inspired oxygen
PCAS: Post-cardiac arrest syndrome
PCI: Percutaneous coronary intervention
ROSC: Return of spontaneous circulation
SOFA: Sequential Organ Failure Assessment
TH: Therapeutic hypothermia
